# Supplementary material for: The Evolution and Characterization of the RNA Interference Pathways in Lophotrochozoa
Source: Genome Biol Evol. 2024 May 7;16(5):evae098. doi: 10.1093/gbe/evae098 (PMC11114477; doi:10.1093/gbe/evae098)
Supplement: evae098_Supplementary_Data [file evae098_supplementary_data.zip › Supplementary_materials.zip!/selected_miRNAs/NoGe_selected_miRNAs/mirdeep_pdf_files/NMRB01000076.1_Notospermus_geniculatus_strain_Ushimado_scaffold76,_whole_genome_shotgun_sequence_2805.pdf]

Provisional ID : NMRB01000076.1_Notospermus_geniculatus_strain_Ushimado_scaffold76,_whole_genome_shotgun_sequence_2805
Score total : 769.4
Score for star read(s) : 3.9
Score for read counts : 764.9
Score for mfe : 1.3
Score for randfold : 0
Score for cons. seed : -0.6
Total read count : 1512
Mature read count : 1506
Loop read count : 0
Star read count : 6

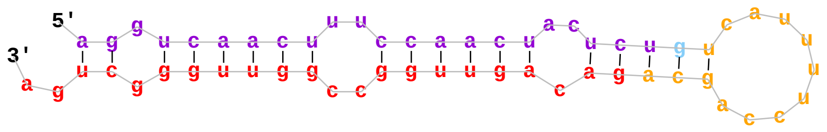

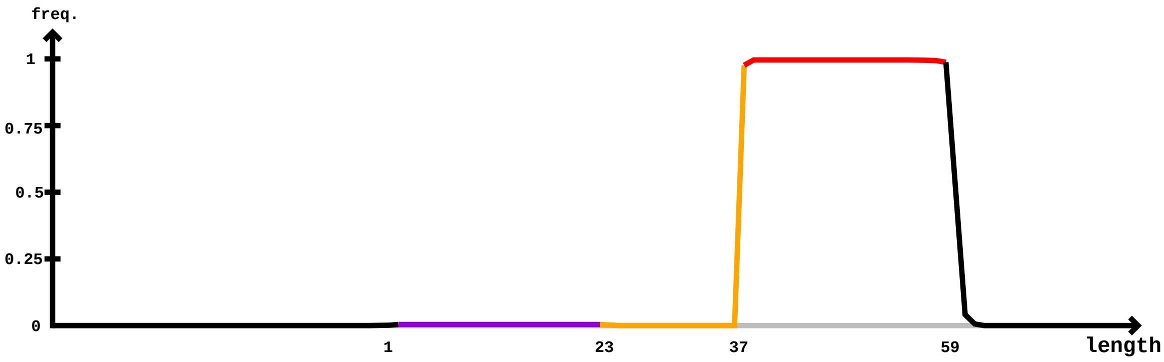

Star
Mature

```
5'- guugugagggucaaagugaaugguuugugacauaaggucaacuuuccaacuacucugucauuuuccagcagacaguuggccgguugggcugauguuuacagaucagugcau  -3'   obs
    guugugagggucaaagugaaugguuugugacauaaggucaacuuuccaacuacucugucauuuuccagcagacaguuggccgguugggcugauguuuacagaucagugcau        exp
    ...............(((..(((((((((((((.((.((((((..((((((..(((((.........))))).))))))..)))))).)).))).)))))))))).)))..  reads   mm   sample
    ......................................................................gacaguuggccgguugggcu.....................  2   0   Fm3
    ......................................................................gacaguuggccgguuggAcug....................  1   1   Fm3
    ......................................................................gacaguuggccgguuggCcuga...................  3   1   Fm3
    ......................................................................Uacaguuggccgguugggcuga...................  1   1   Fm3
    ......................................................................gacaguuggccgguugAgcuga...................  4   1   Fm3
    ......................................................................gacaguuggccgguuggAcuga...................  3   1   Fm3
    ......................................................................gacaguuggcGgguugggcuga...................  2   1   Fm3
    ......................................................................gacaguuggccgguuggUcuga...................  1   1   Fm3
    ......................................................................gacaguuggcAgguugggcuga...................  1   1   Fm3
    ......................................................................Nacaguuggccgguugggcuga...................  1   1   Fm3
    ......................................................................gacaguuggccggAugggcuga...................  1   1   Fm3
    ......................................................................gacagAuggccgguugggcuga...................  1   1   Fm3
    ......................................................................gacaguuggccgguugggAuga...................  1   1   Fm3
    ......................................................................gacaguuggccgguugCgcuga...................  3   1   Fm3
    ......................................................................gCcaguuggccgguugggcuga...................  1   1   Fm3
    ......................................................................gacaguuggGcgguugggcuga...................  1   1   Fm3
    ......................................................................gacaguuggccgguugggcuga...................  524   0   Fm3
    ......................................................................gacaguuggccgguuUggcuga...................  2   1   Fm3
    ......................................................................gacaguuggccgguugggcugU...................  1   1   Fm3
    ......................................................................gacaguuggccgguugggcugaA..................  16   1   Fm3
    ......................................................................gacaguuggccgguugggcugUu..................  1   1   Fm3
    ......................................................................gacaguuggccgguugggcugau..................  5   0   Fm3
    ......................................................................gacaguuggccgguugggcugauA.................  4   1   Fm3
    .......................................................................acaguuggccgguugggcuga...................  10   0   Fm3
    .......................................................................acaguuggccgguugggGuga...................  1   1   Fm3
    .......................................................................acaguuggccgguugggcugau..................  1   0   Fm3

    ......................................................................gacaguuggccgguugggcuga...................  34   0   Mm3
    ......................................................................gacaguuggccgguugggcugG...................  1   1   Mm3
    ......................................................................gacaguuggccgguugggcugU...................  3   1   Mm3
    ......................................................................gacaguuggccgguugggcugaA..................  2   1   Mm3
    ......................................................................gacaguuggccgguugggcugau..................  6   0   Mm3
    ......................................................................gacaguuggccgguugggcugauU.................  1   1   Mm3
```

```
                                 Star                                  Mature
guugugagggucaaagugaaugguuugugacauaaggucaacuuuccaacuacucugucauuuuccagcagacaguuggccgguugggcugauguuuacagaucagugcau

......................................................................gacaguuggccgguugggcuga...................   203   0   Fm1
......................................................................gacGguuggccgguugggcuga...................   1     1   Fm1
......................................................................gacaguuggUcgguugggcuga...................   1     1   Fm1
......................................................................gacaguuggccgguuggCcuga...................   1     1   Fm1
......................................................................gacaguuggccgguugggcugU...................   2     1   Fm1
......................................................................gacaguuggccgguugggcugG...................   4     1   Fm1
......................................................................gacaguuggccgguugCgcuga...................   1     1   Fm1
......................................................................gacaAuuggccgguugggcuga...................   1     1   Fm1
......................................................................gaAaguuggccgguugggcuga...................   1     1   Fm1
......................................................................gacaguuggccgguugggcuAa...................   1     1   Fm1
......................................................................gacaguuggccgguuggAcuga...................   3     1   Fm1
......................................................................gacaguuggccgguugggcugaA..................   5     1   Fm1
......................................................................gacaguuggccgguugggcugau..................   23    0   Fm1
......................................................................gacaguuggccgguugggcugauA.................   4     1   Fm1
......................................................................gacaguuggccgguugggcugauU.................   8     1   Fm1
.......................................................................acaguuggccgguugggcuga...................   2     0   Fm1
.......................................................................acaguuggccgguugggcugaA..................   3     1   Fm1

......................................................................gacaguuggccgguugAgcuga...................   1     1   Fm2
......................................................................gacaguuggccgguuggAcuga...................   1     1   Fm2
......................................................................gacaguuggccgguuggUcuga...................   2     1   Fm2
......................................................................gacaguuggccgguugggcugG...................   10    1   Fm2
......................................................................gacaguuggccgguugggcuga...................   242   0   Fm2
......................................................................gacaguuggcGgguugggcuga...................   1     1   Fm2
......................................................................Uacaguuggccgguugggcuga...................   1     1   Fm2
......................................................................gacaguuggccgguugggcugaA..................   25    1   Fm2
......................................................................gacaguuggccgguugggcugau..................   11    0   Fm2
......................................................................gacaguuggccgguugggcugauA.................   6     1   Fm2
......................................................................gacaguuggccgguugggcugauU.................   15    1   Fm2
.......................................................................acaguuggccgguugggcuga...................   5     0   Fm2
.......................................................................acaguuggccgguugggcugau..................   3     0   Fm2

......................................................................gacaguuggccgguugggcug....................   5     0   Mm1
......................................................................gacaguuggccgguugggGug....................   1     1   Mm1
......................................................................gacaguuggccgguugggcuga...................   85    0   Mm1
......................................................................gacaguuggccgguuggAcuga...................   1     1   Mm1
......................................................................gacaguuggccgguugUgcuga...................   1     1   Mm1
......................................................................gacaguuggccgguugggcugU...................   2     1   Mm1
......................................................................gacaguuggccgguugCgcuga...................   1     1   Mm1
......................................................................gacaguuggccgguuggUcuga...................   1     1   Mm1
......................................................................gacaguuggccgguugggcugau..................   2     0   Mm1
......................................................................gacaguuggccgguugggcugaG..................   2     1   Mm1
......................................................................gacaguuggccgguugggcugaA..................   14    1   Mm1
......................................................................gacaguuggccgguugggcugauU.................   6     1   Mm1
......................................................................gacaguuggccgguugggcugaAgu................   9     1   Mm1

................................uaaggucaacuuuccaacuacucu.......................................................   1     0   Mm2
.................................aaggucaacuuuccaacuacucu.......................................................   1     0   Mm2
..................................aggucaacuuuccaacuacucu.......................................................   4     0   Mm2
......................................................................gacaguuggccgguuggg.......................   1     0   Mm2
......................................................................gacaguuggccgguugggc......................   1     0   Mm2
......................................................................gacaguuggccgguugggcug....................   2     0   Mm2
......................................................................gacaguuggccgguugggcugU...................   2     1   Mm2
......................................................................gacaguuggccgguugggcuga...................   107   0   Mm2
......................................................................gacaguuggcAgguugggcuga...................   1     1   Mm2
......................................................................gacaguuggccgguugggcugG...................   4     1   Mm2
......................................................................gacaguuggccgguugggcugau..................   6     0   Mm2
......................................................................gacaguuggccgguugggcugaA..................   10    1   Mm2
......................................................................gacaguuggccgguugggcugUu..................   1     1   Mm2
......................................................................gacaguuggccgguugggcugaG..................   1     1   Mm2
......................................................................gacaguuggccgguugggcugauA.................   4     1   Mm2
......................................................................gacaguuggccgguugggcugauU.................   5     1   Mm2
.......................................................................acaguuggccgguugggcuga...................   4     0   Mm2
.......................................................................acaguuggccgguugggcugau..................   1     0   Mm2
```
